# Development and evaluation of a recombinase-aided amplification-lateral flow dipstick assay for rapid and sensitive detection of *Mycoplasma synoviae*

**DOI:** 10.3389/fcimb.2026.1920471

**Published:** 2026-09-16

**Authors:** Shu Wang, Yingkai Zhu, Yuxin Wang, Fang Wang, Kexun Lian, Xuemei Sun, Zexuan Wang, Xinghong Ding, Yali Han, Ruinan Wang, Yaocheng Cui, Huiyong Xuan

**Affiliations:** 1 College of Animal Science and Technology, Shihezi University, Shihezi, Xinjiang, China; 2 Xinjiang Taikun Group Co., Ltd. Innovation Research Institute, Xinjiang Taikun Group Co., Ltd., Changji, Xinjiang, China

**Keywords:** lateral flow dipstick, Mycoplasma synoviae, nucleic acid detection, rapid diagnosis, recombinase-aided amplification

## Abstract

**Background:**

*Mycoplasma synoviae* (MS) is a critical avian pathogen responsible for enormous economic losses in global poultry production. Timely prevention and control of MS epidemics rely heavily on rapid, sensitive and accurate diagnostic approaches, while conventional detection methods remain time-consuming and complicated. Therefore, we developed a visual on-site detection system based on recombinase-aided amplification combined with lateral flow dipstick for MS (RAA-LFD-MS assay).

**Methods:**

According to the design rules for RAA primers and exo-probes, specific primers and a probe were designed targeting the conserved region of the MS *vlhA* gene. A recombinant plasmid containing the target fragment was constructed as the positive standard. After primer–probe screening, key reaction parameters including primer concentration, probe concentration, reaction temperature, and incubation time were systematically optimized. The analytical specificity, sensitivity, and repeatability of the established assay were fully evaluated. To further evaluate the clinical performance of our RAA-LFD-MS assay, a total of 100 clinical specimens suspected of MS infection were tested in parallel using this newly established method, conventional polymerase chain reaction (PCR), and quantitative real-time PCR (qPCR).

**Results:**

The RAA reaction was completed within 20 min at a constant temperature of 37 °C, and amplification products were visually read within 3–5 min using a lateral flow dipstick without sophisticated equipment. The total detection time, including the DNA extraction step, is completed within 40 min. Methodological validation revealed that the RAA-LFD assay exhibited high specificity, with no cross-reactivity observed against other common avian pathogens. The assay also showed satisfactory repeatability and high sensitivity, with a limit of detection of 2.63×10^0^ copies/μL. In the clinical sample evaluation, the coincidence rate between RAA-LFD and PCR was 96%, while the agreement between RAA-LFD and qPCR reached 100%.

**Conclusion:**

In summary, the established RAA-LFD assay is rapid, user-friendly, highly specific, and sensitive. To further improve the RAA-LFD-MS assay for routine clinical and field use, future efforts should prioritize the optimization of rapid DNA extraction, accessible temperature-control devices, and reagent lyophilization.

## 1 Introduction

The *Mycoplasma synoviae* (MS) is one of the most important avian pathogens, causing substantial economic losses to the poultry industry worldwide (Han et al., 2023). Infections caused by this pathogen are clinically characterized by respiratory inflammation, synovitis, and osteoarthritis (Stipkovits and Kempf, 1996). Although MS infection rarely causes acute mortality in poultry, it leads to infectious synovitis, respiratory disease, growth retardation, reduced egg production, and the production of misshapen eggs (Gharibi et al., 2018; Kursa et al., 2019; de Jong et al., 2021). Breeding flocks infected with MS can transmit the pathogen vertically to their offspring, and horizontal transmission can also occur via direct respiratory contact (Feberwee et al., 2022). Meanwhile, infection reduces host immunity and significantly increases the risk of secondary infections with other pathogens, thereby elevating flock morbidity and mortality (Xu et al., 2021; Zhang et al., 2023), resulting in severe economic losses for the poultry industry. Therefore, accurate and effective detection is a prerequisite for the identification, prevention, and control of this disease.

At present, conventional laboratory techniques for MS detection mainly include pathogen isolation and identification, serological assays, and molecular biological methods (Wang et al., 2024a). Although pathogen isolation and culture represent the gold standard for clinical diagnosis of MS, this approach is time-consuming, typically requiring 3–4 weeks to complete the entire isolation and culture process, making it difficult to meet the demand for rapid diagnosis (Feberwee et al., 2005). In addition, *in vitro* culture of MS imposes stringent requirements on medium components (Sun et al., 2017), further increasing the difficulty of pathogen isolation. Serological detection techniques rely on specific antibodies produced by the host after infection; however, such antibodies can usually be detected effectively only 2–3 weeks post-infection, resulting in obvious limitations for the diagnosis of early infection (Zhang et al., 2024). PCR and qPCR assays are rapid and efficient, yielding accurate diagnostic results within several hours, making them particularly suitable for emergency epidemic control. These molecular methods have become the mainstream techniques for molecular detection of MS infection (Yadav et al., 2022; Wang et al., 2023). Nevertheless, such molecular assays require expensive thermal cyclers and strict professional laboratory conditions.

To overcome the above drawbacks, isothermal nucleic acid amplification techniques suitable for MS detection have been developed, such as polymerase spiral reaction (PSR) and loop-mediated isothermal amplification (LAMP) (Kursa et al., 2015; Wu et al., 2019). However, these two techniques still have shortcomings: the amplification reaction takes 40–60 min, and LAMP requires the design of six complementary primers, which increases the difficulty of primer design (Kursa et al., 2015). Therefore, the development and promotion of a simple and rapid detection technique for MS are of great practical significance and urgent necessity. Isothermal amplification techniques have consequently gained increasing attention, particularly RPA and RAA. A representative study constructed an RT-RPA-CRISPR/Cas13 lateral flow visualization system for rapid detection of avian infectious bronchitis virus, which achieved high specificity and sensitivity within 50 min without sophisticated laboratory instruments, offering a reliable paradigm for developing point-of-care detection methods for poultry pathogens (Khudeir et al., 2024). Among these, RAA offers a distinct advantage over RPA, as its recombinase is derived from more accessible and cost-effective sources, thereby exhibiting greater potential for widespread application and translation (Mao et al., 2023).

RAA is an emerging isothermal nucleic acid amplification technology (Tian et al., 2025). Its basic principle is that recombinase in the reaction system binds with primers to form complexes and searches for target sequences on the template; single-stranded DNA-binding proteins assist in unwinding the double-stranded DNA template, enabling DNA polymerase to initiate sequence amplification (Chen et al., 2018; Li et al., 2023). This technique can rapidly complete nucleic acid amplification at a constant temperature of 37–42°C, and the entire reaction usually takes only 15–30 min (Chen et al., 2018). When a specific probe is incorporated into the reaction system, amplification products can be conveniently and visually detected using a lateral flow dipstick (LFD) (Zheng et al., 2021). LFD is a point-of-care testing device based on immunochromatography or nucleic acid hybridization, which drives sample migration via capillary action (Parolo et al., 2020). Target analysis is rapidly achieved by color development at the test line (T-line) and control line (C-line) (de Puig et al., 2017). The operation is simple, and results can be obtained within 5–10 min without sophisticated equipment. LFD has been widely used in clinical pathogen detection, food safety screening, and other scenarios (Gao et al., 2020; Wang et al., 2024b). In recent years, the RAA-LFD technique has been successfully applied to the detection of various human and animal pathogens (Wang et al., 2020; Zuo et al., 2025).

This study established a rapid RAA-LFD-MS assay with high specificity and sensitivity, providing a novel technical tool for clinical diagnosis and field detection of MS.

## 2 Materials and methods

### 2.1 Materials

#### 2.1.1 Reagents and equipment

RAA nucleic acid amplification reagents (lateral flow dipstick format, Cat. No. B8203C5) and single-target HybriDetect lateral flow dipsticks (rainbow type, Cat. No. JY0201) were purchased from Nanjing Warbio Biotechnology Co., Ltd. (Nanjing, China). Gel recovery kits (Cat. No. DC301-01), DNA purification kits (Cat. No. RC313-01), plasmid mini-extraction kits (Cat. No. DC201-01), and the pMD19-T vector (Cat. No. 3271) were obtained from Vazyme Biotech Co., Ltd. (Nanjing, China). A mixed solution of phenol:chloroform:isoamyl alcohol (25:24:1, v/v/v, Cat. No. P1012) and Biowest agarose (Cat. No. 111860) were purchased from Xinjiang Hengchao Biotechnology Co., Ltd. (Xinjiang, China).

*Escherichia coli* DH5α competent cells (Cat. No. DF1001) were acquired from Tsingke Biotechnology Co., Ltd. (Beijing, China). Ampicillin (Cat. No. S17018) was purchased from Yuanye Bio-Technology Co., Ltd. (Shanghai, China). 2×Universal Blue SYBR Green qPCR Master Mix (Cat. No. G3326-01), DNA molecular weight markers (Cat. No. G3360-01), and phosphate-buffered saline (PBS, Cat. No. G0002) were obtained from Servicebio Technology Co., Ltd. (Wuhan, China). Gold View nucleic acid stain (Cat. No. GV0010) was purchased from Beijing Aoboxing Tuoda Technology Co., Ltd. (Beijing, China). 2×Es Taq PCR Master Mix (with dye, Cat. No. CW0690H) and nuclease-free deionized water (ddH_2_O Cat. No. CW0690H) were obtained from ComWin Biotech Co., Ltd. (Beijing, China).

A water bath incubator (Cat. No. WB100-1) was purchased from Qunan Scientific Instrument Co., Ltd. (Zhejiang, China). A high-speed desktop centrifuge (Cat. No. MIKRO 120) was obtained from Nanjing Haidixi Equipment Co., Ltd. (Nanjing, China). A conventional PCR thermal cycler (Cat. No. 2720 Thermal Cycler) was purchased from Thermo Fisher Scientific Inc. (Waltham, MA, USA). A real-time fluorescence quantitative PCR (qPCR) system (Cat. No. Gentier 96R) was obtained from Tianlong Science and Technology Co., Ltd. (Xi’an, China). A gel imaging system (Cat. No. Gel Doc EZ) was purchased from Bio-Rad Laboratories Inc. (Hercules, CA, USA). A horizontal electrophoresis system (Cat. No. SPW-DM) was obtained from Servicebio Technology Co., Ltd. (Wuhan, China).

#### 2.1.2 Bacterial strains and clinical samples

Strains adopted in this study were all clinical isolates archived in our laboratory, comprising four MS strains (MS-1, MS-2, MS-3, MS-4), *Mycoplasma gallisepticum* (MG), *Mycoplasma bovis* (Mb), *Pasteurella multocida* (Pm), *Mannheimia haemolytica* (Mh), *Staphylococcus aureus* (Sa), *Salmonella* spp., *Escherichia coli*, *Bacillus subtilis*, *Enterococcus faecium* and *Enterococcus faecalis*. Closely related Mycoplasma species were selected to validate the method’s discriminatory performance for MS, while common opportunistic pathogens and commensal bacteria of chicken origin were introduced specifically to simulate complex clinical microbial niches and to evaluate potential cross-reactivity. A total of 100 throat swab specimens were gathered from chickens with suspected MS infection at commercial broiler farms in Shihezi, Xinjiang Uygur Autonomous Region, China. All specimens were preserved at −80 °C prior to subsequent experimental manipulation (Confer, 1993; Woo and Kim, 2006; Barnes et al., 2008; Nguyen et al., 2015; El Zowalaty et al., 2023; Matos et al., 2024; Zhou et al., 2025).

### 2.2 Methods

#### 2.2.1 Nucleic acid extraction

Genomic DNA from MS, MG, Mb, Pm, Mh, Sa, *Salmonella* spp., *Escherichia coli*, *Bacillus subtilis*, *Enterococcus faecium*, and *Enterococcus faecalis* was extracted using a DNA purification kit according to the manufacturer’s instructions. All DNA templates were stored at −20 °C until use.

#### 2.2.2 Preparation of plasmid standard

The *vlhA* gene of MS was amplified by PCR using the forward primer 5′-TTGCAATGTCAGAAGAAGCT-3′ and the reverse primer 5′-GCTTGGAATAATGAAGATTC-3′. The 20 μL PCR mixture contained 10 μL of 2×Es Taq PCR Master Mix (with dye), 6 μL of ddH_2_O, 1 μL of each forward and reverse primer (10 μmol/L), and 2 μL of DNA template. The thermal cycling protocol was set as follows: pre-denaturation at 95 °C for 5 min; 32 cycles of denaturation at 95 °C for 30 s, annealing at 55 °C for 30 s, and extension at 72 °C for 30 s; and a final extension at 72 °C for 7 min.

The amplified *vlhA* fragment was purified using a gel recovery kit and then cloned into the pMD19-T vector. The 10 μL ligation mixture included 4 μL of the purified *vlhA* amplicon, 1 μL of pMD19-T vector, and 5 μL of Solution I ligase. Ligation was performed at 4 °C for 12 h. Competent Escherichia coli DH5α cells stored at −80 °C were thawed on ice. Then, 10 μL of the ligation product was gently mixed with DH5α cells in a laminar flow hood, followed by incubation on ice for 30 min, heat shock at 42 °C for 90 s, and immediate chilling on ice for 3 min. Subsequently, 1 mL of antibiotic-free LB broth was added, and the cells were incubated at 37 °C with shaking at 200 rpm for 1 h. After centrifugation at low speed for 1 min, the supernatant was discarded, and 200 μL of the bacterial suspension was spread onto LB agar plates supplemented with ampicillin. The plates were incubated upside down at 37 °C for 12–16 h until single colonies appeared. Single colonies were picked and inoculated into 5 mL of LB broth containing ampicillin, followed by incubation at 37 °C with shaking at 220 rpm for 12 h. Positive clones were confirmed by colony PCR using the universal pMD19-T vector primers M13F (5′-CGCCAGGGTTTTCCCAGTCACGAC-3′) and M13R (5′-AGCGGATAACAATTTCACACAGGA-3′). The PCR program was as follows: 95 °C for 5 min; 30 cycles of 95 °C for 30 s, 55 °C for 30 s, and 72 °C for 1 min; and a final extension at 72 °C for 7 min. Positive recombinant plasmids were sent to Sangon Biotech Co., Ltd. (Shanghai, China) for Sanger sequencing, and the sequences were aligned using the NCBI BLAST tool. Recombinant plasmid DNA (pMD19-*vlhA*) was extracted using a plasmid mini kit and stored at −20 °C. After verification by PCR and sequencing, the plasmid concentration was determined using an ultra-micro spectrophotometer, and the plasmid copy number was calculated using the following formula (Equation 1):

(1) $$\text{Plasmid copy number (copies/μL)}=\left[\text{plasmid concentration (g/μL)}\times 6.02\times 10^{23}\right]/\left[\text{plasmid length (bp)}\times \text{660 g/mol}\right]$$

#### 2.2.3 Primer design and screening

Multiple *Mycoplasma synoviae vlhA* gene sequences (GenBank accession Nos. AF035624.1, HQ682228.2, HQ326483.1, MN304940.1, and HQ326481.1) were retrieved and subjected to multiple sequence alignment to screen the highly conserved regions. Based on the conserved fragment located at 1037–1606 bp of the reference sequence AF035624.1 and the reaction mechanism of recombinase-aided amplification (RAA), four pairs of specific primers were designed using Oligo 7.0 software. All primers and probes were synthesized by Sangon Biotech Co., Ltd. (Shanghai, China), and the detailed sequences are listed in **Table 1**. The RAA reaction was performed in a 50 μL volume according to the instructions of the basic RAA nucleic acid amplification kit. Briefly, the reaction premix consisted of 25 μL of Buffer A, 13.5 μL of ddH_2_O, 2 μL of forward primer (10 μmol/L), and 2 μL of reverse primer (10 μmol/L). The mixture was thoroughly blended in a clean PCR tube and then transferred into a reaction tube containing lyophilized RAA powder. Next, 5 μL of template DNA was added, and 2.5 μL of Buffer B was dropped onto the inner wall of the tube cap. The tube was tightly capped, gently inverted and flicked 5–6 times for complete mixing, briefly centrifuged for 10 s, and then incubated at 39 °C for 30 min in a constant-temperature water bath. After incubation, 50 μL of phenol:chloroform:isoamyl alcohol (25:24:1, v/v/v) was added to the mixture. After thorough homogenization, the tube was centrifuged at 12,000 rpm for 5 min. The supernatant was collected and analyzed by 20 g/L agarose gel electrophoresis to screen the optimal primer pair.

**Table 1:** RAA primer sequences.

Primer name	Genes	Sequence (5'→3')	Product size(bp)
MS-F1	*vlhA*	CCAACTCAAGCAGCTACTCCAACAACAACG	178
MS-R1		AAGTGTCATTTAATGATTTTAGATAAGAAG	
MS-F2		ATAGAGCACAACACGAAGCTGCAAATAGAC	167
MS-R2		TCTAACTTCATCTCAAGAAATTGTAGGATT	
MS-R2b		Biotin-TCTAACTTCATCTCAAGAAATTGTAGGATT	
MS-F3		AAGAAGCTGCAGCAGAAGCAGAAACTCAAA	183
MS-R3		AGTATAGTGTACGACCGTCAACTGGTTTGT	
MS-F4		TTAACGGTGATAACCCAACAGAAAAAACAG	150
MS-R4		CTCTATTGTTTTCGGCATTATTTGCTACGT	
MS-Probe 1		FAM-TTAAAGTTAACCAAGATAAATTAAGCCTTGTAGC[THF]GAGCAGCTAACAAAA-C3spacer	
MS-Probe 2		FAM-AGAACAGTGATTTAAAGTTAACCAAGATAA[THF]TTAAGCCTTGTAGCAGAG-C3spacer	

#### 2.2.4 Probe design and screening

In this study, primer screening, probe design and modification, as well as the construction of the RAA-LFD detection system, were performed following the methods previously established in our laboratory (Xuan et al., 2026). Following primer screening, the optimal primer pair was determined to be MS F2/MS R2, which generated a single, bright, and expected-sized amplicon in agarose gel electrophoresis during RAA. Subsequently, the reverse primer was labeled with biotin at the 5′-end and designated as MS R2b. Meanwhile, two detection probes were designed according to the instructions of the RAA-LFD kit (**Table 1**). Each probe was labeled with a FAM fluorescent group at the 5′-end, contained a tetrahydrofuran (THF) residue in the middle, and was blocked with C3 phosphorylation at the 3′-end. To evaluate potential false-positive signals caused by primer–probe complexes on the dipstick, each probe was validated using the RAA-LFD assay in the absence of MS DNA template.

#### 2.2.5 Establishment of the RAA-LFD reaction system

Referring to previous studies, the following RAA reaction system was established in this study (Xuan et al., 2026). Based on the optimal primer–probe combination, a 50 μL RAA reaction system was established according to the manufacturer’s instructions for the lateral flow dip-stick-based RAA nucleic acid amplification kit. The reaction premix contained 25 μL of Buffer A, 12.9 μL of ddH_2_O, 2 μL of forward primer (2 μmol/L), 2 μL of reverse primer (2 μmol/L), and 0.6 μL of probe (2 μmol/L). The mixture was thoroughly mixed in a clean PCR tube and transferred into a reaction tube containing lyophilized RAA powder. Then, 5 μL of DNA template was added, and 2.5 μL of Buffer B was dropped onto the inner wall of the tube cap. The tube was tightly sealed, gently inverted and flicked 5–6 times for complete homogenization, briefly centrifuged for 10 s, and incubated at 39 °C for 30 min in a constant-temperature water bath. After incubation, 50 μL of the reaction mixture was diluted 1:6 in phosphate-buffered saline (PBS), and the lateral flow dipstick was immediately immersed into the diluted sample for visualization.

#### 2.2.6 Optimization of RAA-LFD reaction conditions

The optimal primer pair was serially diluted, and forward and reverse primers were added into the reaction system at stock concentrations of 0.5, 1, 1.5, 2, 3, and 4 μmol/L, respectively, with other components kept constant, to determine the optimal primer concentration. Based on the optimal primer concentration, the probe was diluted to stock concentrations of 0.5, 1, 1.5, 2, 3, and 4 μmol/L to screen the optimal probe concentration. Using the determined optimal primer and probe concentrations, reactions were performed at 25, 30, 35, 37, 39, and 41 °C for 30 min to identify the optimal reaction temperature. Finally, the reaction time was set to 10, 15, 20, 25, and 30 min to determine the optimal amplification duration.

#### 2.2.7 Specificity assay

The analytical specificity of the established RAA-LFD assay was evaluated using genomic DNA extracted from MS, MG, Mb, Pm, Mh, Sa, Salmonella, E. coli, B. subtilis, E. faecium, and E. faecalis as templates. Nuclease-free ddH_2_O served as the negative control. All reactions were conducted under the optimized optimal conditions, and the results were observed using the lateral flow dipstick.

#### 2.2.8 Sensitivity assay

The recombinant positive plasmid standard pMD19-*vlhA* was 10-fold serially di-luted to yield concentrations ranging from 10^7^ to 10^0^ copies/μL, which were used as templates to evaluate the analytical sensitivity of RAA-LFD, conventional PCR, and qPCR. The RAA-LFD assay was performed under the optimized conditions described above. For PCR, the same primer set as in RAA-LFD was used. The 20 μL PCR mixture contained 10 μL of PCR Mix, 6 μL of ddH_2_O, 1 μL of each forward and reverse primer (10 μmol/L), and 2 μL of DNA template. The thermal cycling conditions were as follows: pre-denaturation at 95 °C for 5 min; 32 cycles of denaturation at 95 °C for 30 s, annealing at 60 °C for 30 s, and extension at 72 °C for 30 s; and a final extension at 72 °C for 7 min.

For qPCR, SYBR Green qPCR Master Mix was employed with the same primer set as in RAA. The 10 μL qPCR mixture consisted of 5 μL of qPCR Mix, 0.2 μL of each forward and reverse primer (10 μM), 3.6 μL of nuclease-free water, and 1 μL of DNA template. The qPCR program was set as follows: pre-denaturation at 95 °C for 30 s, followed by 40 cycles of denaturation at 95 °C for 15 s, annealing at 60 °C for 10 s, and extension at 72 °C for 30 s.

#### 2.2.9 Repeatability assay

The established RAA-LFD assay was subjected to intra-assay and inter-assay repeatability tests using three concentrations of recombinant plasmid standards (2.63×10^4^, 2.63×10³, and 2.63×10² copies/μL) in triplicate, and the corresponding amplification results were analyzed.

#### 2.2.10 Clinical sample detection

A total of 100 throat swab samples were collected from chickens suspected of being infected with MS in three commercial broiler farms in Shihezi City, Xinjiang Uygur Autonomous Region, China. These chickens exhibited typical clinical signs including lameness, dyspnea, joint swelling, and growth retardation. None of the chickens had been vaccinated against MG or MS. No isolation and culture of *Mycoplasma* was performed on all clinical specimens; genomic DNA was directly extracted from the original throat swab samples without pre-culture treatment before molecular detection. All samples were detected in parallel by the established RAA-LFD-MS assay, conventional PCR, and qPCR.

## 3 Results

### 3.1 RAA-LFD assay strategy

Prior to establishing the detection system, multiple *vlhA* gene sequences of MS (accession numbers: AF035624.1, HQ682228.2, HQ326483.1, MN304940.1, HQ326481.1) were retrieved from NCBI for multiple sequence alignment to screen highly-conserved regions. The fragment spanning 1037–1606 bp of reference sequence AF035624.1 was selected as the target region. On this basis, specific RAA primer pairs and Nfo probes carrying THF-modified sites were designed (**Figures 1a, b**).Throat swab and joint fluid specimens collected from suspected infected chickens were processed with commercial DNA extraction kits to obtain genomic templates within 15 min (**Figure 1c**). The prepared RAA reaction mixture underwent isothermal amplification in a water bath at 37 °C for 20 min, during which Nfo nuclease recognized and cleaved the THF residue on the probe to release dual FAM/biotin-labeled amplicons (**Figure 1d**). The reacted solution was diluted 1:6 with PBS buffer and loaded onto lateral flow strips for immunochromatographic reaction: gold nanoparticles conjugated with anti-FAM antibodies bound the FAM tags of target products, immobilized biotin ligands on the test line captured biotin moieties, and the control line confirmed valid flow. Simultaneous bands on the test and control lines represented positive results, while a single band only on the control line indicated negative detection (**Figure 1e**).

**Figure 1:**
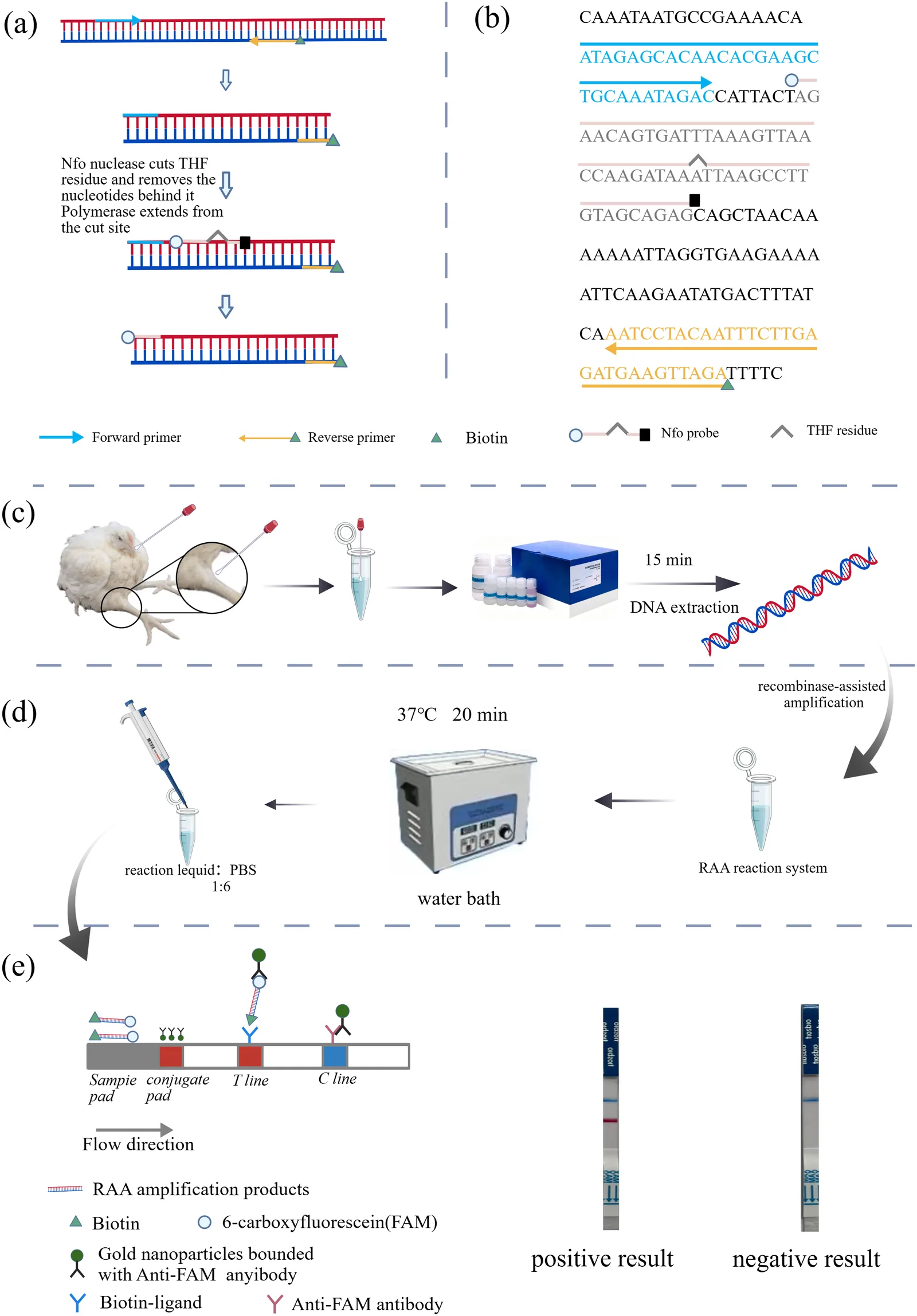
Schematic overview of the MS RAA‑LFD detection assay. **(a)** Principle of the RAA‑LFD amplification reaction. The Nfo‑containing probe binds to the target sequence; Nfo nuclease cleaves the THF residue of the probe, removes downstream nucleotides, and polymerase extends from the cleavage site to generate dual‑labeled amplicons. **(b)** Multiple‑sequence alignment of the *vlhA* gene, in which conserved regions were selected for RAA primer and probe design. **(c)** Sample processing and nucleic acid extraction. Throat swab and joint fluid specimens are collected from chickens suspected of MS infection, and genomic DNA is extracted by commercial kits within 15 min to serve as amplification template. **(d)** Isothermal RAA amplification. The RAA reaction system is incubated in a water‑bath at 37 °C for 20 min, and the amplified products are diluted 1:6 with PBS buffer. **(e)** Lateral‑flow strip detection and result interpretation. After sample loading, gold nanoparticles conjugated with anti‑FAM antibodies interact with FAM‑labeled amplicons; the T‑line captures biotin‑modified products, and the C‑line validates successful liquid flow. Visible bands on both T‑line and C‑line represent positive results, whereas only the C‑line band indicates negative results.

### 3.2 Primer and probe screening

Four primer pairs were screened by basic RAA amplification, and the products were analyzed using 20 g/L agarose gel electrophoresis. The results showed that the primer pair MS F2/MS R2 yielded a single, intense, and clear amplicon with the fewest non-specific bands (**Figure 2a**). Probe screening revealed that both candidate probes generated clear positive test bands on lateral flow dipsticks; however, MS-Probe 1 showed non-specific false-positive signals in the absence of template DNA. Consequently, the primer pair MS F2/MS R2 and MS-Probe 2 were selected for subsequent experiments (**Figure 2b**).

**Figure 2:**
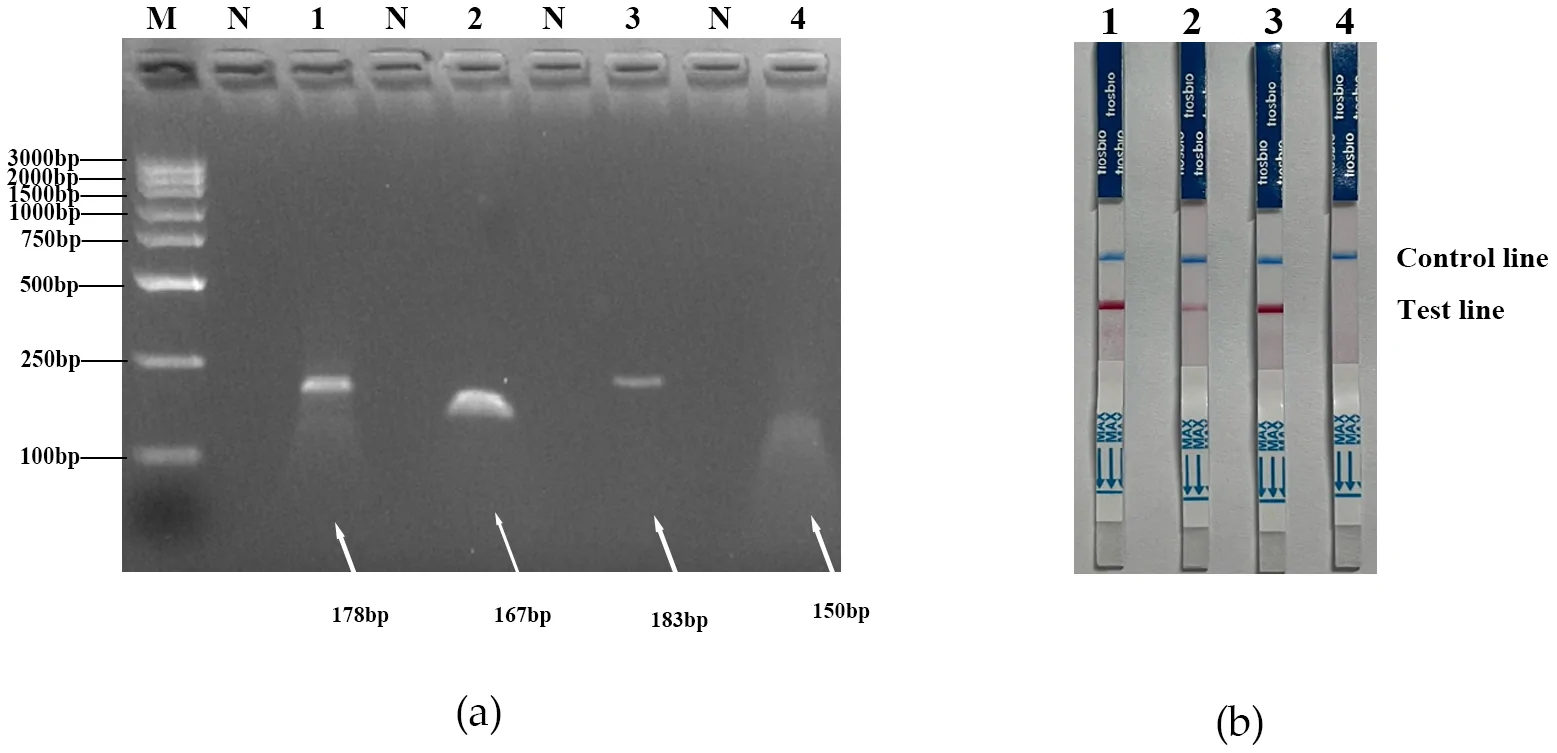
Screening of MS primers and probes. **(a)** Grayscale image for MS primer screening. Lanes 1–4: Positive amplification products of primer pairs 1 to 4; M: DNA marker DL 3000; N: Blank control. **(b)** Screening of primer-probe combinations. 1: MS F2/MS R2/MS-Probe 1 combination; 2: Negative control for MS F2/MS R2/MS-Probe 1 combination; 3: MS F2/MS R2/MS-Probe 2 combination; 4: Negative control for MS F2/MS R2/MS-Probe 2 combination.

### 3.3 Conditions optimization of RAA reaction primer concentration, temperature and time

To establish a robust RAA-LFD detection system, key reaction parameters including primer concentration, probe concentration, reaction temperature, and incubation time were optimized through gradient experiments under consistent reaction conditions. The primer concentration was optimized while keeping all other reaction parameters constant. As shown in **Figure 3a**, distinct target bands were observed at all primer concentrations ranging from 0.5 to 4 μM, and stronger band intensity was achieved at 2–4 μM. Thus, 2 μM was determined as the optimal primer concentration for subsequent assays. The optimal probe concentration of the RAA-LFD system was further evaluated. As illustrated in **Figure 3b**, visible bands were detected at probe concentrations from 0.5 to 4 μM, with brighter signals observed at 1–4 μM. Therefore, the lowest effective concentration of 1 μM was chosen as the optimal probe concentration. The optimal reaction temperature was also investigated. As presented in **Figure 3c**, positive bands appeared at temperatures ranging from 30 to 41 °C, with stronger signals at 37–41 °C. Consequently, 37 °C was selected as the optimal reaction temperature. Furthermore, the optimal reaction time of the RAA-LFD assay was verified (**Figure 3d**). Clear target bands were observed within 10–30 min, and the band intensity reached the highest level at 20–30 min. Thus, the shorter and more efficient time of 20 min was adopted as the optimal reaction duration.

**Figure 3:**
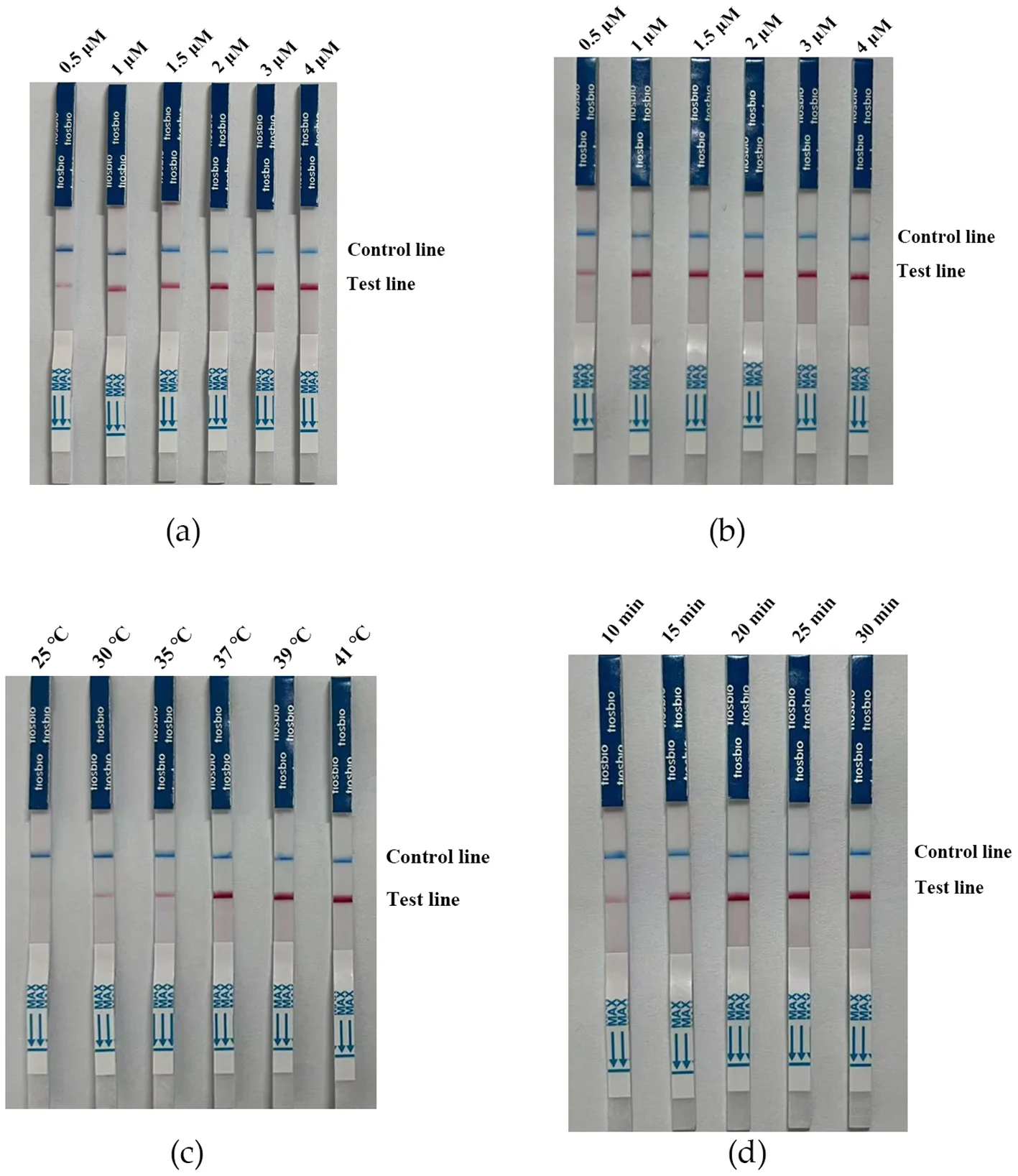
Optimization results of MS RAA-LFD detection reaction conditions. **(a)** Optimization results of primer concentration. **(b)** Optimization results of probe concentration. **(c)** Optimization results of reaction temperature. **(d)** Optimization results of reaction time.

### 3.4 Specificity test

To evaluate the analytical specificity of the established RAA-LFD-MS assay, genomic DNAs of various common poultry pathogens and environmental bacteria were used as templates, and nuclease-free water was set as the negative control for cross-reaction detection under optimized reaction conditions. The results demonstrated that the target band was successfully amplified from DNA of all MS strains, whereas no visible band was observed for DNA of other pathogenic bacteria (**Figure 4a**). Collectively, these findings indicated that the established RAA-LFD-MS assay exhibited high specificity, with no cross-reactivity with other common pathogenic microorganisms.

**Figure 4:**
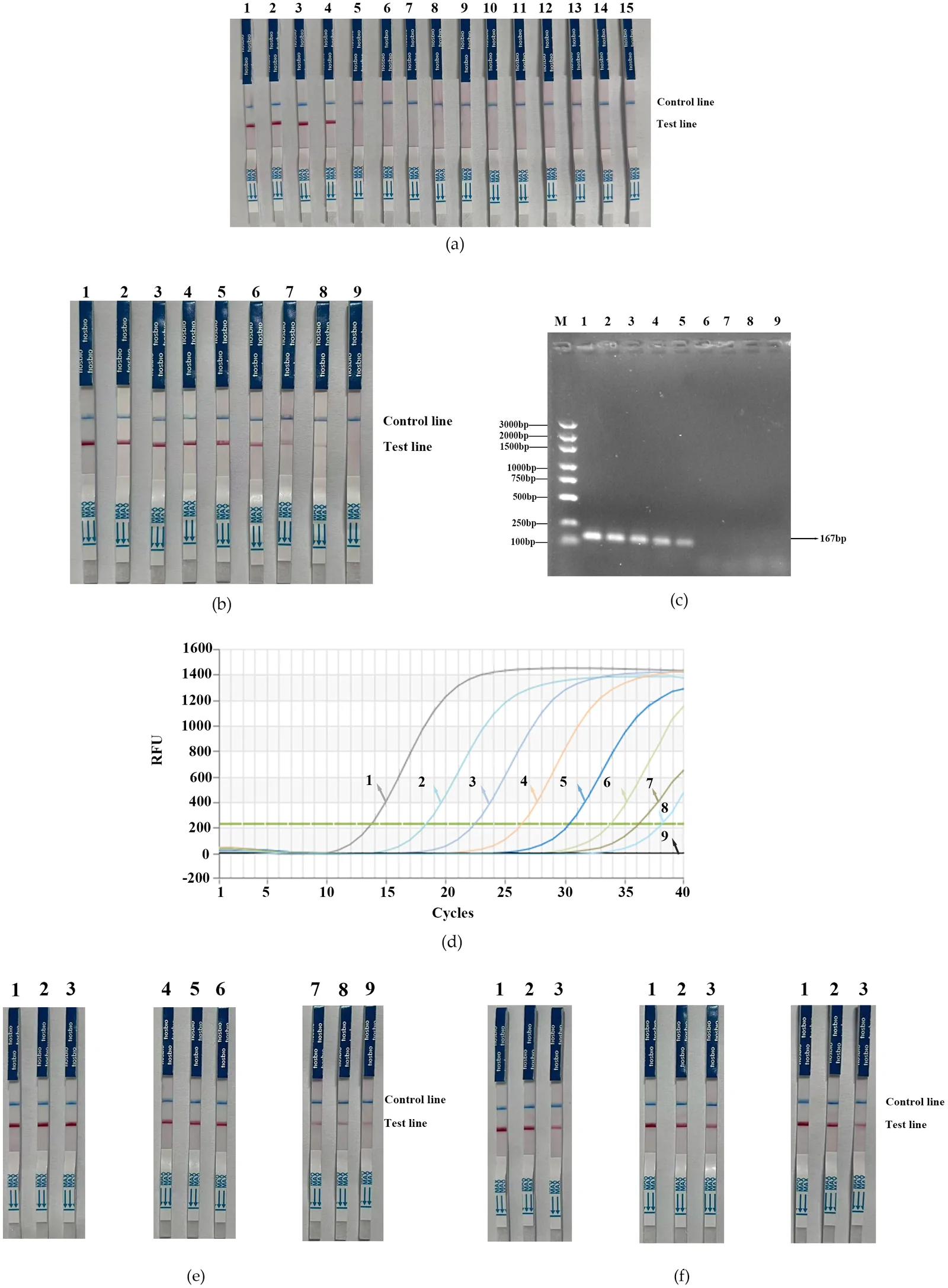
Performance evaluation results of MS RAA-LFD. **(a)** Specificity test results of MS RAA-LFD, 1–4: MS RAA-LFD detection results, 5–14: RAA-LFD detection results for MG, Mb, Pm, Mh, Sa, Salmonella spp., Escherichia coli, Bacillus subtilis, Enterococcus faecium and Enterococcus faecalis. 15: Negative control. **(b)** Sensitivity test results of MS RAA-LFD. **(c)** Sensitivity test results of MS PCR. **(d)** Sensitivity test results of MS qPCR. 1–8: MS DNA template concentrations ranging from 2.63×10^7^ to 2.63×10^0^ copies/µl. 9: Negative control. M: Standard molecular weight is DL 3000. **(e)** Intra-assay repeatability test results of MS RAA-LFD, 1–3: DNA template concentration at 2.63×10^4^ copies/µL. 4–6: DNA template concentration at 2.63×10^3^ copies/µL. 7–9: DNA template concentration at 2.63×10^2^ copies/µL. **(f)** Inter-assay repeatability test results of MS RAA-LFD, 1: DNA template concentration at 2.63×10^4^ copies/µL. 2: DNA template concentration at 2.63×10^3^ copies/µL. 3: DNA template concentration at 2.63×10^2^ copies/µL.

### 3.5 Comparison of the sensitivity of RAA, PCR and qPCR

To assess the analytical sensitivity of the developed method, serially diluted recombinant plasmid standards were used as templates. The detection performance of RAA-LFD, conventional PCR, and qPCR was compared using identical primer sequences and standard reaction procedures for each system. In the present study, the analytical sensitivities of the RAA-LFD, conventional PCR, and qPCR assays were compared. The results revealed that the limits of detection (LODs) of the three methods were 2.63×10^0^ copies/μL, 2.63×10³ copies/μL, and 2.63×10^0^ copies/μL, respectively (**Figures 4b–d**).

### 3.6 Repeatability test

The intra-assay and inter-assay repeatability of the RAA-LFD-MS assay was evaluated using high, medium, and low concentrations of recombinant plasmid standards. Amplification consistency among parallel and independent experiments was analyzed to verify method stability.

The results of the intra-assay repeatability test are shown in **Figure 4e**. The target bands were successfully amplified at all tested concentrations, and the intensities of the amplified bands were highly consistent for each replicate at the same concentration.

As illustrated in **Figure 4f**, the inter-assay repeatability test revealed that the target fragments were steadily amplified across three independent experiments at all concentrations, and the band intensities were highly uniform among different replicate groups.

### 3.7 Detection of clinical samples

Three methods, namely RAA, PCR, and qPCR, were used for simultaneous detection of 100 clinical samples suspected of MS infection. The results showed that the positive rate of RAA detection was 48% (48/100), while the positive rates of PCR and qPCR were 44% (44/100) and 48% (48/100), respectively. The detection coincidence rate between RAA and PCR was 96%, and that between RAA and qPCR was 100% (**Table 2**). In addition, all 44 samples identified as positive by PCR were also positive in RAA detection, resulting in a positive percent agreement of 100%. Among the 56 samples determined as negative by PCR, 52 were also negative in RAA detection, with a negative percent agreement of 92.9%. Compared with the PCR method, the kappa value of the RAA assay established in this study was 0.92 (**Table 3**). These results indicate that the RAA assay established in this study can be used for the detection of MS in clinical samples.

**Table 2:** Detection results of clinical samples by different methods (n=100).

Methods	Positive cases	Negative cases	Positive rate (%)	Coincidence rate (%)
RAA-LFD	48	52	48	–
PCR	44	56	44	96
qPCR	48	52	48	100

**Table 3:** Performance comparison of RAA-LFD and PCR methods for detecting MS in clinical samples (n=100).

RAA‑LFD assay		PCR		Performance characteristic				
		Positive	Negative	Positive percent agreement (%)	Negative percent agreement (%)	PPV (%)	NPV (%)	Kappa
RAA-LFD	Positive	44	4	100	92.9	91.7	100	0.92
	Negative	0	52					

RAA-LFD, Recombinase-Aided Amplification combined with Lateral Flow Device. PPV, Positive Predictive Value. NPV, Negative Predictive Value.

## 4 Discussion

MS represents a dominant avian pathogen that imposes substantial economic burdens on the global poultry industry by inducing respiratory disorders, synovitis, and reduced growth performance (Feberwee et al., 2022; Yadav et al., 2022). Rapid, reliable, and field-deployable detection of MS is critical for timely intervention, epidemiological surveillance, and disease control in both commercial farms and grassroots veterinary settings. In the present study, a RAA-LFD-MS assay was successfully developed and validated for rapid and visual detection of MS.

The analytical sensitivity of the RAA-LFD assay reached a limit of detection (LOD) of 2.63×10^0^ copies/μL, which was equivalent to that of qPCR and approximately 1000-fold higher than that of conventional PCR. This remarkable sensitivity positioned the RAA-LFD assay as a highly reliable molecular tool for the detection of MS at low pathogen loads, including subclinical infections and early-stage infections. In comparison with previously reported isothermal amplification techniques for MS detection, such as LAMP and PSR, which typically require 40–60 min for completion and involve complex primer sets (Kursa et al., 2015; Wu et al., 2019), the RAA-LFD assay accomplished amplification within 20 min at a constant temperature of 37 °C and achieved visual readout within 3–5 min using lateral flow dipsticks. This improvement in speed and simplicity rendered the RAA-LFD assay more applicable for rapid and on-site diagnosis. Moreover, the assay eliminated the requirement for thermal cyclers and professional laboratory conditions, which are indispensable for PCR and qPCR (Shahid et al., 2013; Dijkman et al., 2017), thereby expanding its utility in resource-limited settings and field surveillance.

The specificity assessment demonstrated that the established RAA-LFD assay exclusively amplified the target fragment from MS genomic DNA, whereas no cross-reactivity was observed against a panel of common avian pathogens, including MG, Mb, Pm, Mh, Sa, *Salmonella* spp., *Escherichia coli*, *Bacillus subtilis*, *Enterococcus faecium*, and *Enterococcus faecalis*. This high specificity can be attributed to the rigorous design of primers and a probe targeting the conserved *vlhA* gene of MS, which minimized non-specific annealing and off-target amplification. Similar findings have been documented in previously established isothermal amplification assays targeting avian pathogens, where conserved gene-based primer-probe design contributed to exceptional analytical specificity (Wang et al., 2020; Zuo et al., 2025). The elimination of cross-reactivity is particularly critical in clinical settings, where MS infections frequently occur alongside other bacterial or viral pathogens (Bolha et al., 2013; Roussan et al., 2015), thereby ensuring diagnostic accuracy and reducing false-positive results.

The repeatability assessment revealed that the intra-assay and inter-assay evaluations yielded highly consistent amplification bands at three distinct concentrations of the recombinant plasmid standard, indicating favorable reproducibility and stability of the established RAA-LFD assay. This reproducibility was comparable to that of previously established RAA-based assays for avian and livestock pathogens (He et al., 2021; Zheng et al., 2021), confirming the reliability and robustness of the assay for routine diagnostic applications. The stability and reproducibility of the assay were further enhanced by the optimization of key reaction parameters, including primer concentration, probe concentration, reaction temperature, and incubation duration, which maximized amplification efficiency and reduced intra- and inter-assay variations.

In the evaluation of 100 clinical samples collected from commercial broiler farms, the RAA-LFD assay exhibited a positive rate of 48%, which was consistent with that of qPCR (48%) and higher than that of conventional PCR (44%). The coincidence rate between RAA-LFD and PCR was 96%, whereas the agreement between RAA-LFD and qPCR reached 100%. The kappa value between the RAA-LFD assay and PCR was 0.92, indicating almost perfect agreement. These results demonstrated that the RAA-LFD assay possessed high diagnostic accuracy and clinical applicability for the detection of MS in clinical specimens. In addition, the positive percent agreement of the RAA-LFD assay relative to PCR was 100%, and the negative percent agreement was 92.9%, further confirming its reliability for clinical diagnosis. Collectively, the RAA-LFD assay could serve as a reliable and practical alternative to PCR and qPCR for the rapid detection of MS in clinical and field settings.

Compared with traditional MS diagnostic methods, the RAA-LFD assay established in this study exhibited multiple superiorities. Pathogen isolation and culture, the gold standard for MS diagnosis, requires 3–4 weeks and imposes stringent requirements for medium composition and operational techniques (Sun et al., 2017; Xue et al., 2017), rendering it unsuitable for rapid diagnosis and field surveillance. Serological assays, such as ELISA, depend on specific antibodies produced 2–3 weeks post-infection, leading to delayed detection and potential cross-reactivity (Zhang et al., 2024; Liu et al., 2025). Although PCR and qPCR are rapid and sensitive, they rely on expensive thermal cyclers and specialized laboratory conditions, limiting their field application (Shahid et al., 2013; Dijkman et al., 2017). In contrast, the RAA-LFD assay integrates the advantages of isothermal amplification and lateral flow dipstick-based visual detection, featuring rapidity, simplicity, low equipment dependence, and visual readout. The entire assay could be completed within 25 min (20 min amplification plus 3–5 min visual readout) at a constant temperature of 37 °C without sophisticated instruments, making it suitable for auxiliary rapid screening in resource-limited primary-level laboratories and poultry farm sites.

In summary, the established RAA-LFD-MS assay is a rapid, user-friendly, highly specific, sensitive, and reproducible method for the visual detection of MS. Compared with conventional PCR and qPCR, this assay does not rely on large sophisticated thermal cyclers, and only simple heating equipment is required, making it a robust and practical auxiliary screening tool for MS testing in resource-limited grassroots veterinary laboratories and poultry farms. Notably, future efforts to advance the RAA-LFD-MS assay toward routine clinical and field applications should focus on the optimization of rapid DNA extraction protocols, portable and easy-to-operate temperature-control instruments, and robust lyophilization processes for reagent preservation.

## Data Availability

The original contributions presented in the study are included in the article/**Supplementary Material**. Further inquiries can be directed to the corresponding authors.
