# Correction: Development and evaluation of a recombinase-aided amplification-lateral flow dipstick assay for rapid and sensitive detection of *Mycoplasma synoviae*

**DOI:** 10.3389/fcimb.2026.2013735

**Published:** 2026-10-12

**Authors:** Shu Wang, Yingkai Zhu, Yuxin Wang, Fang Wang, Kexun Lian, Xuemei Sun, Zexuan Wang, Xinghong Ding, Yali Han, Ruinan Wang, Yaocheng Cui, Huiyong Xuan

**Affiliations:** 1 College of Animal Science and Technology, Shihezi University, Shihezi, Xinjiang, China; 2 Xinjiang Taikun Group Co., Ltd. Innovation Research Institute, Xinjiang Taikun Group Co., Ltd., Changji, Xinjiang, China

**Keywords:** lateral flow dipstick, Mycoplasma synoviae, nucleic acid detection, rapid diagnosis, recombinase-aided amplification

An incorrect **Funding** statement was provided. The correct **Funding** statement reads:

“This study was funded by the project entitled Establishment and Preliminary Performance Evaluation of a Multiplex Detection Platform for Avian *Mycoplasma* Based on Recombinase-Aided Amplification Combined with Lateral Flow Strip Assay (2026D01B129); Corps Finance and Administration [2024] No.97–2024 Talent Development Fund-Tianchi Talents (the Second Batch) for Young Doctors (CZ004309); Corps Finance and Administration [2024] No.97–2024 Talent Development Fund-Tianchi Talents (the Second Batch) for Young Doctors (CZ004312); the University-level High-Level Talent Project of Shihezi University in Xinjiang Province, China (RCZK202455); University-level High-Level Talent Project of Shihezi University in Xinjiang Province, China (RCZK202454).”

The original version of this article has been updated.

