# Supplementary material for: Development and evaluation of a recombinase-aided amplification-lateral flow dipstick assay for rapid and sensitive detection of Mycoplasma synoviae
Source: Front Cell Infect Microbiol. 2026 Sep 16;16:1920471. doi: 10.3389/fcimb.2026.1920471 (PMC13623553; doi:10.3389/fcimb.2026.1920471)
Supplement: Supplementary file 1 [file DataSheet1.zip › Supplementary files/MS figure/MS figure legends.doc]

Figure.1 Screening of MS primers and probes. a: Grayscale image for MS primer screening. Lanes 1–4: Positive amplification products of primer pairs 1 to 4; M: DNA marker DL 3000; N: Blank control. b: Screening of primer-probe combinations. 1: MS F2/MS R2/MS-Probe 1 combination; 2: Negative control for MS F2/MS R2/MS-Probe 1 combination; 3: MS F2/MS R2/MS-Probe 2 combination; 4: Negative control for MS F2/MS R2/MS-Probe 2 combination.

Figure. 2 Optimization results of MS RAA-LFD detection reaction conditions. a: Optimization results of primer concentration. b: Optimization results of probe concentration. c: Optimization results of reaction temperature. d: Optimization results of reaction time

 Figure. 3 Performance evaluation results of MS RAA-LFD. a: Specificity test results of MS RAA-LFD, 1–4: MS RAA-LFD detection results, 5–14: RAA-LFD detection results for MG, Mb, Pm, Mh, Sa, Salmonella spp., Escherichia coli, Bacillus subtilis, Enterococcus faecium and Enterococcus faecalis. 15: Negative control. b: Sensitivity test results of MS RAA-LFD. c: Sensitivity test results of MS PCR. d: Sensitivity test results of MS qPCR. 1–8: MS DNA template concentrations ranging from 2.63×10⁷ to 2.63×10⁰ copies/μl. 9: Negative control. M: Standard molecular weight is DL 3000. e: Intra-assay repeatability test results of MS RAA-LFD, 1–3: DNA template concentration at 2.63×10⁴ copies/μL. 4–6: DNA template concentration at 2.63×10³ copies/μL. 7–9: DNA template concentration at 2.63×10² copies/μL. f: Inter-assay repeatability test results of MS RAA-LFD, 1: DNA template concentration at 2.63×10⁴ copies/μL. 2: DNA template concentration at 2.63×10³ copies/μL. 3: DNA template concentration at 2.63×10² copies/μL.

Figure. 4 Workflow diagram of the MS RAA-LFD detection assay. First, throat swabs and joint fluid samples are collected from chickens suspected of Mycoplasma synoviae (MS) infection, followed by genomic DNA extraction to serve as the template for subsequent amplification reactions. Subsequently, the recombinase-aided amplification (RAA) isothermal reaction system is constructed and incubated in a conventional 37°C water bath for 20 minutes. After amplification, the RAA product is diluted 1:6 (v/v, volume/volume) with phosphate-buffered saline (PBS), and the diluted reaction mixture is loaded onto a lateral flow dipstick (LFD). Based on the principle of immunochromatography, the dipstick enables visual detection of amplified products through specific binding between gold-labeled antibodies, the test line (coated with antigen-specific capture antibody 1), and the control line (coated with anti-gold antibody). Finally, the results are interpreted based on band patterns: a positive result is defined as the appearance of both the test line (T line) and the control line (C line), indicating the presence of the target gene in the sample; a negative result is defined as the appearance of only the control line (C line), indicating the absence of the target gene in the sample.
