# Supplementary material for: Development and evaluation of a recombinase-aided amplification-lateral flow dipstick assay for rapid and sensitive detection of Mycoplasma synoviae
Source: Front Cell Infect Microbiol. 2026 Sep 16;16:1920471. doi: 10.3389/fcimb.2026.1920471 (PMC13623553; doi:10.3389/fcimb.2026.1920471)
Supplement: Supplementary file 1 [file DataSheet1.zip › Supplementary files/Supplementary Materials/Supplementary Material 1.doc]

Figure 1. RAA-LFD Results of Partial Clinical Samples. No.1–No.10: Clinical Samples No.1–No.10

Figure 2. RAA-LFD Results of Partial Clinical Samples. No.11–No.20: Clinical Samples No.11–No.20

Figure 3. RAA-LFD Results of Partial Clinical Samples. No.21–No.30: Clinical Samples No.21–No.30

Figure 4. RAA-LFD Results of Partial Clinical Samples. No.31–No.40: Clinical Samples No.31–No.40

Figure 5. RAA-LFD Results of Partial Clinical Samples. No.41–No.50: Clinical Samples No.41–No.50

Figure 6. RAA-LFD Results of Partial Clinical Samples. No.51–No.60: Clinical Samples No.51–No.60

Figure 7. RAA-LFD Results of Partial Clinical Samples. No.61–No.70: Clinical Samples No.61–No.70

Figure 8. RAA-LFD Results of Partial Clinical Samples. No.71–No.80: Clinical Samples No.71–No.80

Figure 9. RAA-LFD Results of Partial Clinical Samples. No.81–No.90: Clinical Samples No.81–No.90

Figure 10. RAA-LFD Results of Partial Clinical Samples. No.91–No.100: Clinical Samples No.91–No.100
