# Supplementary material for: Development and evaluation of a recombinase-aided amplification-lateral flow dipstick assay for rapid and sensitive detection of Mycoplasma synoviae
Source: Front Cell Infect Microbiol. 2026 Sep 16;16:1920471. doi: 10.3389/fcimb.2026.1920471 (PMC13623553; doi:10.3389/fcimb.2026.1920471)
Supplement: Supplementary file 1 [file DataSheet1.zip › Supplementary files/Supplementary Materials/Supplementary Material 2.doc]

Figure 1. PCR Results of Partial Clinical Samples. M: DNA marker DL3000. No.1–No.25: Clinical Samples No.1–No.25

Figure 2. PCR Results of Partial Clinical Samples. M: DNA marker DL3000. No.26–No.50: Clinical Samples No.26–No.50

Figure 3. PCR Results of Partial Clinical Samples. M: DNA marker DL3000. No.51–No75: Clinical Samples No.51–No.75

Figure 4. PCR Results of Partial Clinical Samples. M: DNA marker DL3000. No.76–No.100: Clinical Samples No.76–No.100
