# Supplementary material for: Development and evaluation of a recombinase-aided amplification-lateral flow dipstick assay for rapid and sensitive detection of Mycoplasma synoviae
Source: Front Cell Infect Microbiol. 2026 Sep 16;16:1920471. doi: 10.3389/fcimb.2026.1920471 (PMC13623553; doi:10.3389/fcimb.2026.1920471)
Supplement: Supplementary file 1 [file DataSheet1.zip › Supplementary files/Supplementary Materials/Supplementary Material 3.doc]

Table 1. qPCR Results of Partial Clinical Samples.

| Sample | Ct | Sample | Ct |
| --- | --- | --- | --- |
| 1 | - | 26 | 13.879 |
| 2 | - | 27 | - |
| 3 | - | 28 | - |
| 4 | - | 29 | - |
| 5 | 14.707 | 30 | - |
| 6 | 15.098 | 31 | 14.512 |
| 7 | - | 32 | 14.949 |
| 8 | 15.145 | 33 | 13.691 |
| 9 | 18.426 | 34 | - |
| 10 | 18.965 | 35 | - |
| 11 | 32.465 | 36 | 13.980 |
| 12 | - | 37 | - |
| 13 | - | 38 | 13.965 |
| 14 | 22.441 | 39 | 22.504 |
| 15 | - | 40 | - |
| 16 | - | 41 | 17.254 |
| 17 | - | 42 | 14.160 |
| 18 | - | 43 | 14.238 |
| 19 | 22.832 | 44 | 14.176 |
| 20 | - | 45 | - |
| 21 | - | 46 | 32.520 |
| 22 | - | 47 | - |
| 23 | - | 48 | - |
| 24 | - | 49 | - |
| 25 | 25.723 | 50 | 18.027 |

Table 2. qPCR Results of Partial Clinical Samples.

| Sample | Ct | Sample | Ct |
| --- | --- | --- | --- |
| 51 | - | 76 | 14.043 |
| 52 | - | 77 | 14.043 |
| 53 | 22.371 | 78 | 14.316 |
| 54 | 32.809 | 79 | 14.410 |
| 55 | 21.777 | 80 | - |
| 56 | - | 81 | - |
| 57 | - | 82 | 14.168 |
| 58 | - | 83 | 14.676 |
| 59 | 18.379 | 84 | 13.965 |
| 60 | - | 85 | 18.207 |
| 61 | 14.066 | 86 | 13.378 |
| 62 | - | 87 | 14.550 |
| 63 | 13.512 | 88 | 32.371 |
| 64 | - | 89 | 17.934 |
| 65 | - | 90 | - |
| 66 | 14.496 | 91 | 13.987 |
| 67 | 13.723 | 92 | 14.302 |
| 68 | 13.684 | 93 | 14.298 |
| 69 | - | 94 | 13.254 |
| 70 | - | 95 | - |
| 71 | - | 96 | - |
| 72 | - | 97 | - |
| 73 | - | 98 | - |
| 74 | - | 99 | - |
| 75 | 14.012 | 100 | - |

Figure 1. qPCR Results of Clinical Samples 1–25

Figure 2. qPCR Results of Clinical Samples 26–100
